# Supplementary material for: Full-waveform tomography reveals iron spin crossover in Earth’s lower mantle
Source: Nat Commun. 2024 Mar 4;15:1961. doi: 10.1038/s41467-024-46040-1 (PMC10912123; doi:10.1038/s41467-024-46040-1)
Supplement: Supplementary file 1 — Supplementary Information [file 41467_2024_46040_MOESM1_ESM.pdf]

Supplementary Information for
Full-waveform tomography reveals iron spin crossover in Earth's lower mantle

Laura Cobden[1]*, Jingyi Zhuang[2], Wenjie Lei,[2]†,[3] Renata Wentzcovitch[2,4-7]*, Jeannot Trampert[1], Jeroen Tromp[3]

[1]Department of Earth Sciences, Utrecht University; 3584 CB Utrecht, The Netherlands.

[2]Department of Earth and Environmental Sciences, Columbia University; New York NY 10027, USA.

[3]Department of Geosciences, Princeton University; Princeton NJ 08544, USA

[4]Department of Applied Physics and Applied Mathematics, Columbia University; New York, NY 10027, USA

[5]Lamont Doherty Earth Observatory, Palisades, NY 10964, USA

[6]Data Science Institute, Columbia University; New York, NY 10027, USA

[7]Center for Computational Quantum Physics, Flatiron Institute, New York, NY 10010, USA

*Corresponding authors. Email: l.j.cobden@uu.nl (general enquiries); rmw2150@columbia.edu (*ab initio* calculations)

†Present address: Google Inc.

Contents

Figures S1 to S20
Tables S1 to S4

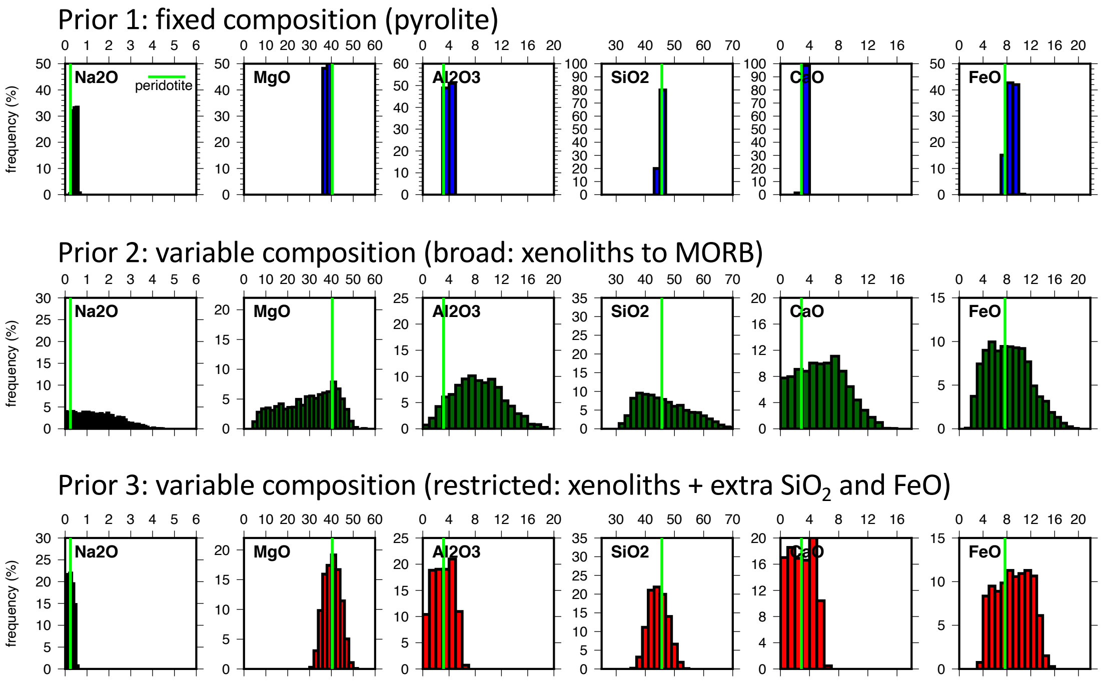

Fig. S1.

Frequency distributions showing the ranges of bulk composition (in wt %) for three different priors. The pale green vertical line shows the values for peridotite[1] for reference.

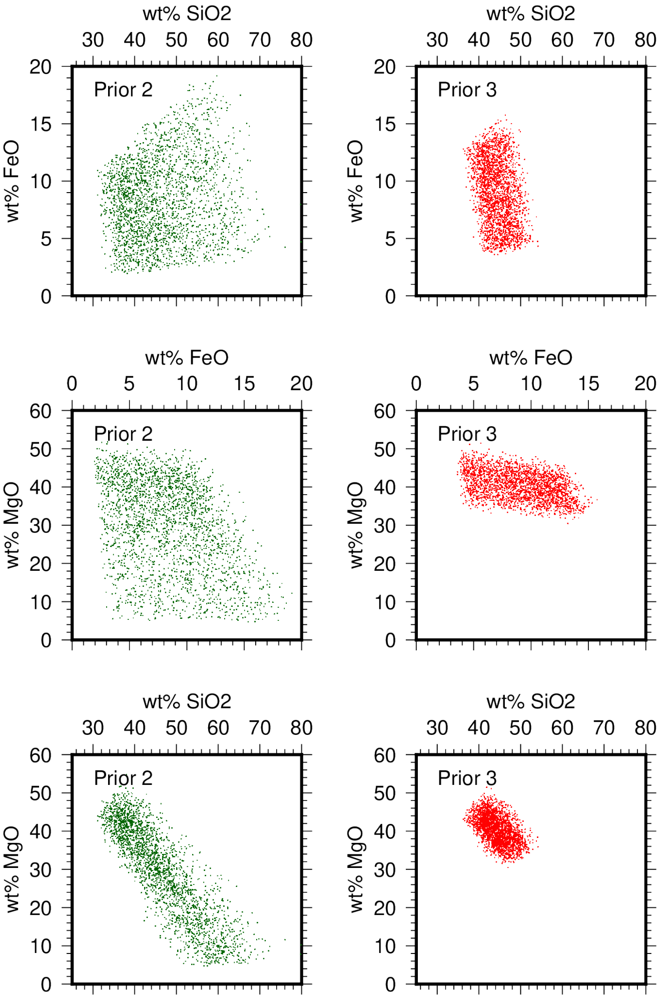

Fig. S2.

Scatter plots showing abundances in wt% of SiO_2, FeO and MgO in the models comprising Priors 2 and 3. There is no significant correlation between FeO and SiO_2 nor between FeO and MgO. There is an anticorrelation between SiO_2 and MgO as these are the most abundant components which means that they cannot simultaneously both be large or small. Note the higher density of points for Prior 3 as the same number of models samples a narrower range in compositional space.

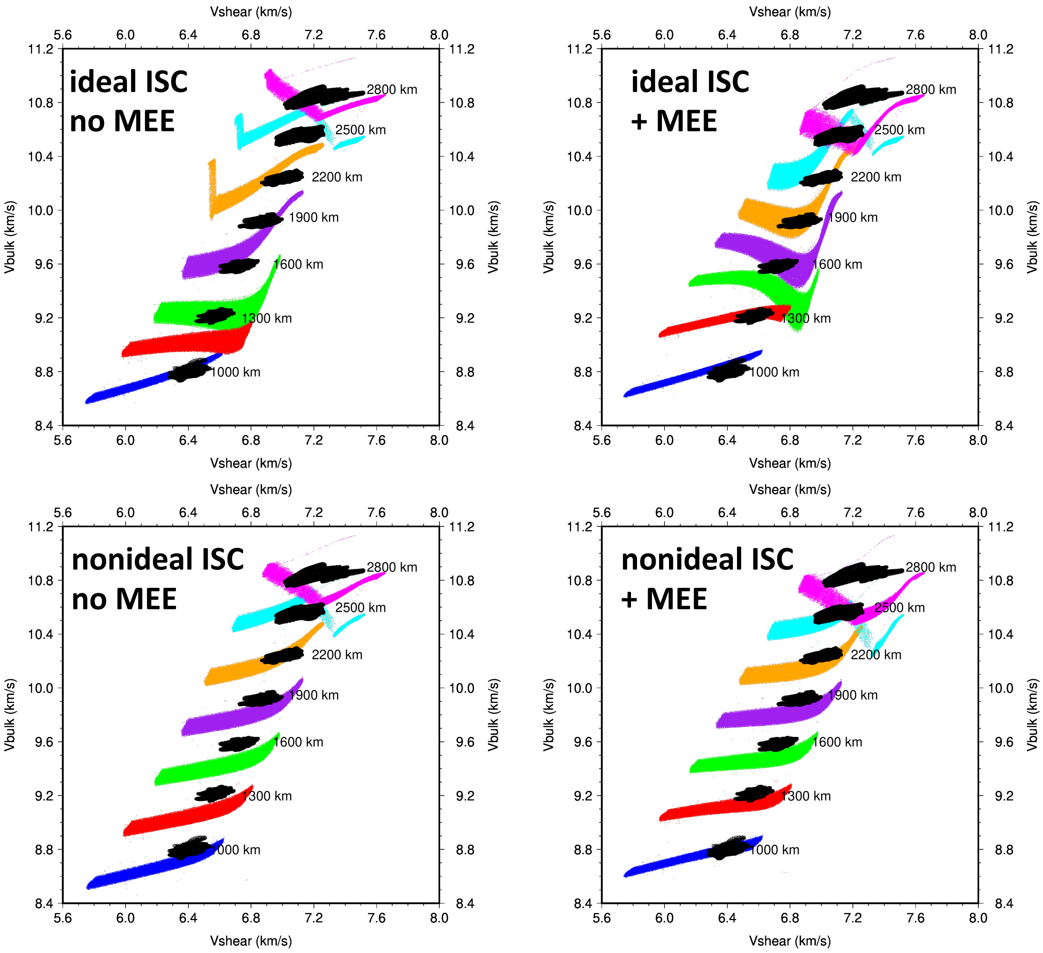

Fig. S3.

Scatter plots showing bulk vs shear wave speed at 300 km depth intervals, for Prior 1 (in rainbow colours) when 4 different theoretical approximations are used to calculate the effect of the iron spin crossover (ISC) in ferropericlase. ideal / nonideal refers to HS-LS mixing and MEE refers to magnetic entropic effects. Black clouds are GLAD-M25.

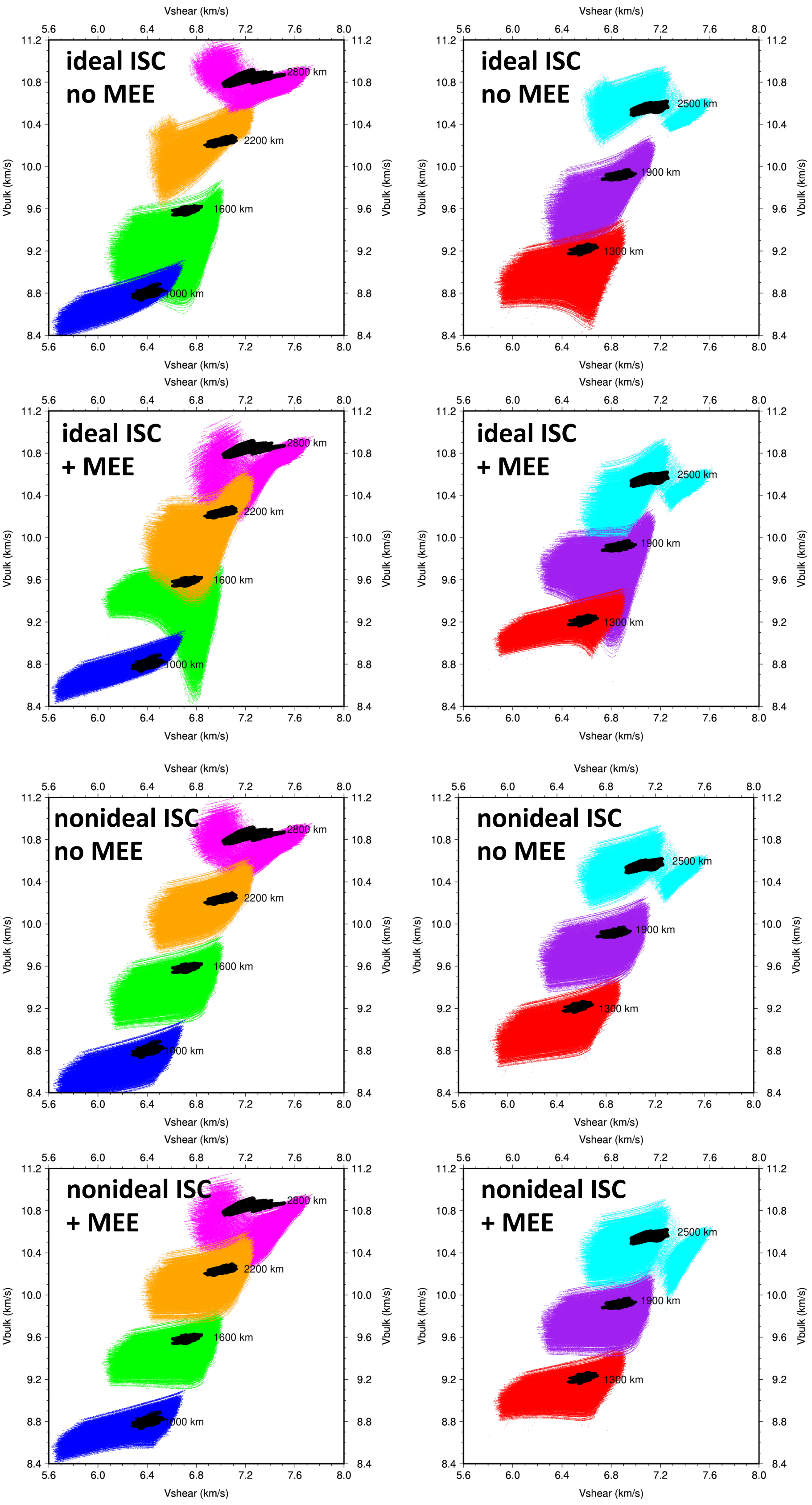

Fig. S4.

Scatter plots showing bulk vs shear wave speed at 300 km depth intervals, for Prior 2 (i.e., variable composition) and 4 different theoretical approximations for the iron spin crossover (ISC) in ferropericlase. ideal / nonideal refers to HS-LS mixing and MEE refers to magnetic entropic effects. Black clouds are GLAD-M25. Coloured clouds represent the thermochemical models and black clouds are GLAD-M25

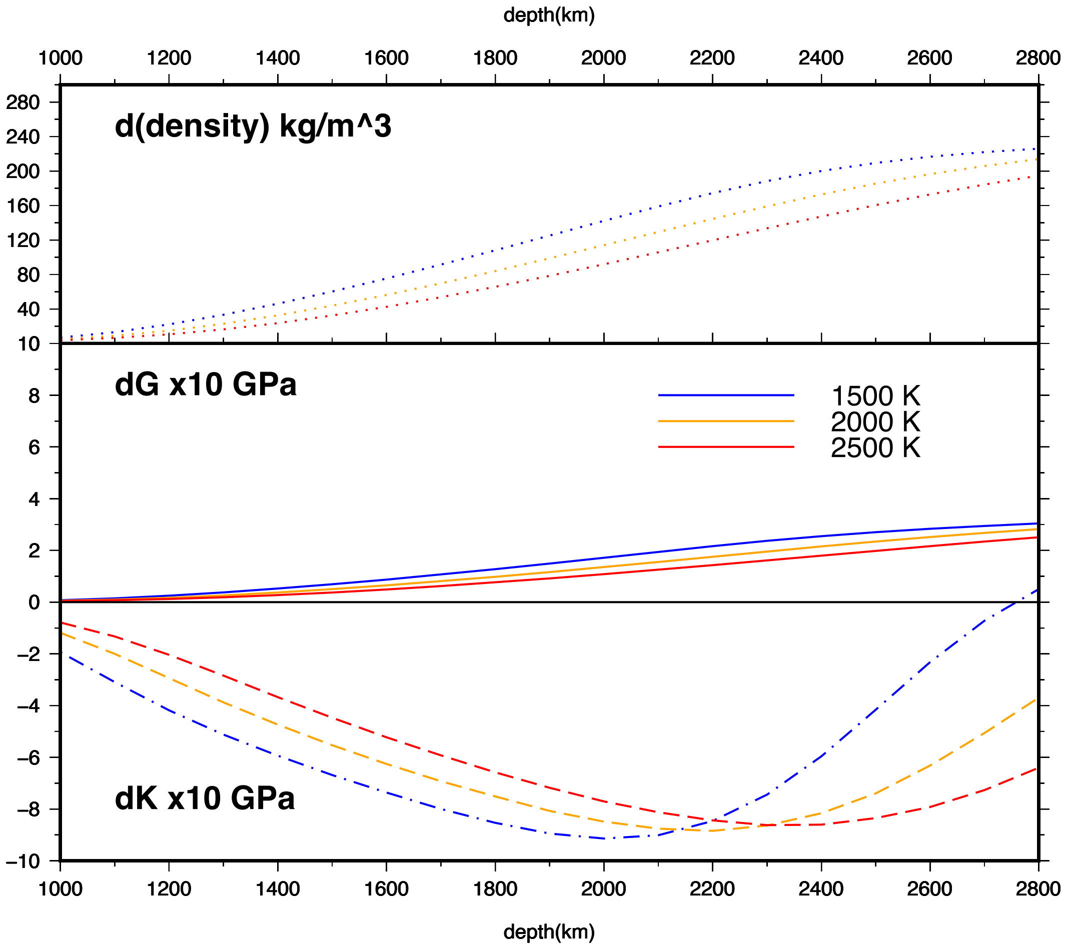

Fig. S5.

Change in density, shear modulus and bulk modulus as a function of depth along 3 different isotherms due to high-to-low spin transition in ferropericlase, including the effects of non-ideal solid solution and magnetic entropy.

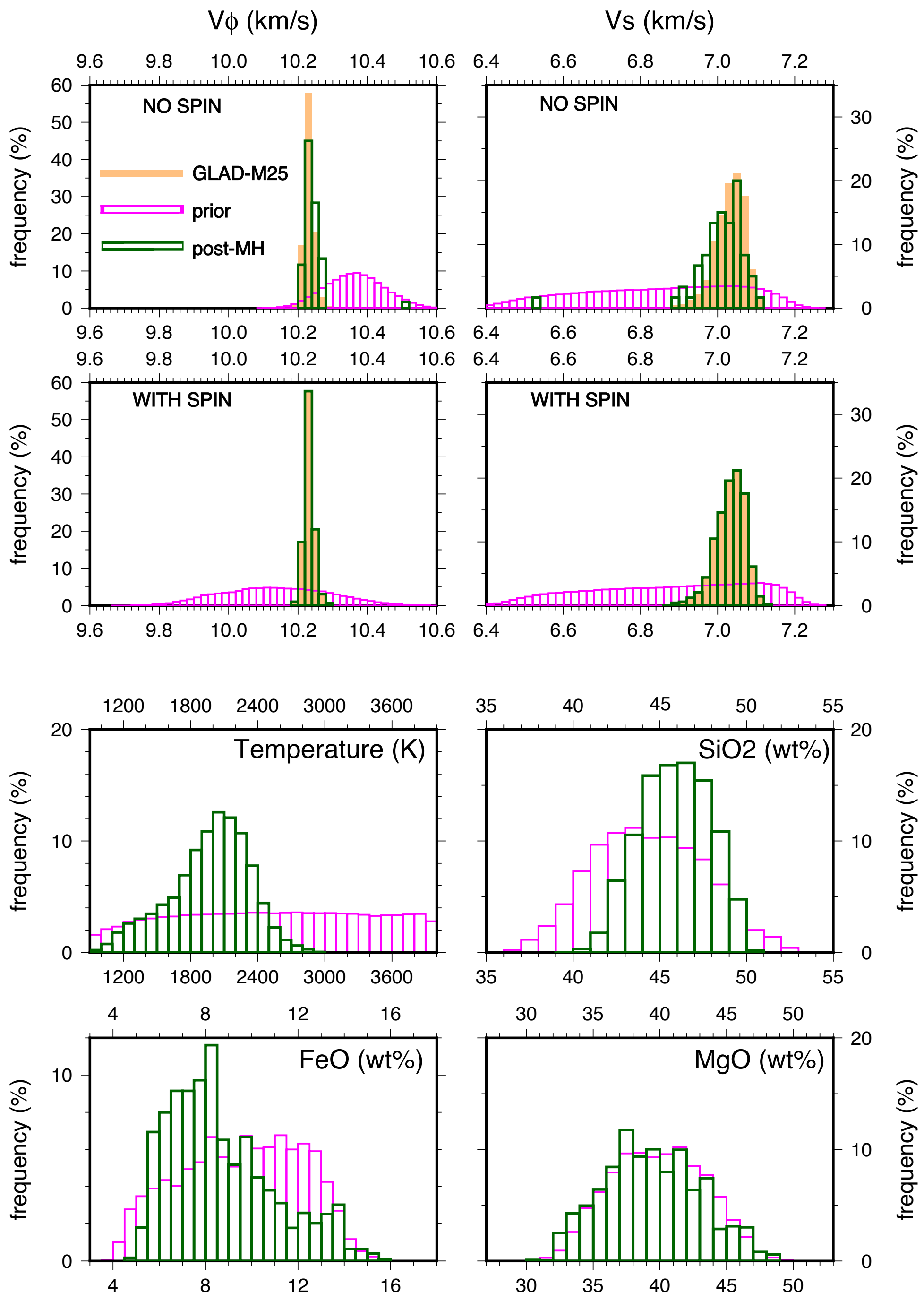

Fig. S6.

Illustration of application of Metropolis-Hastings (MH) algorithm at 2200 km depth. Top two rows: Yellow histograms show the frequency distributions of bulk and shear wave speed in GLAD-M25. Pink histograms show the wave-speed distributions of the prior (Prior 3, Fig. S1), with and without inclusion of effect of a spin transition in ferropericlase. Green histograms show the best fit to GLAD-M25 after applying MH. The degree of overlap between the yellow and green histograms is used to quantify the fit, as plotted in Figure 2. Clearly the fit is better with a spin transition that without. Bottom two rows: distributions of temperature, FeO, SiO_2 and MgO in Prior 3 (pink histograms) versus the remaining subset of models after applying MH (green histograms).

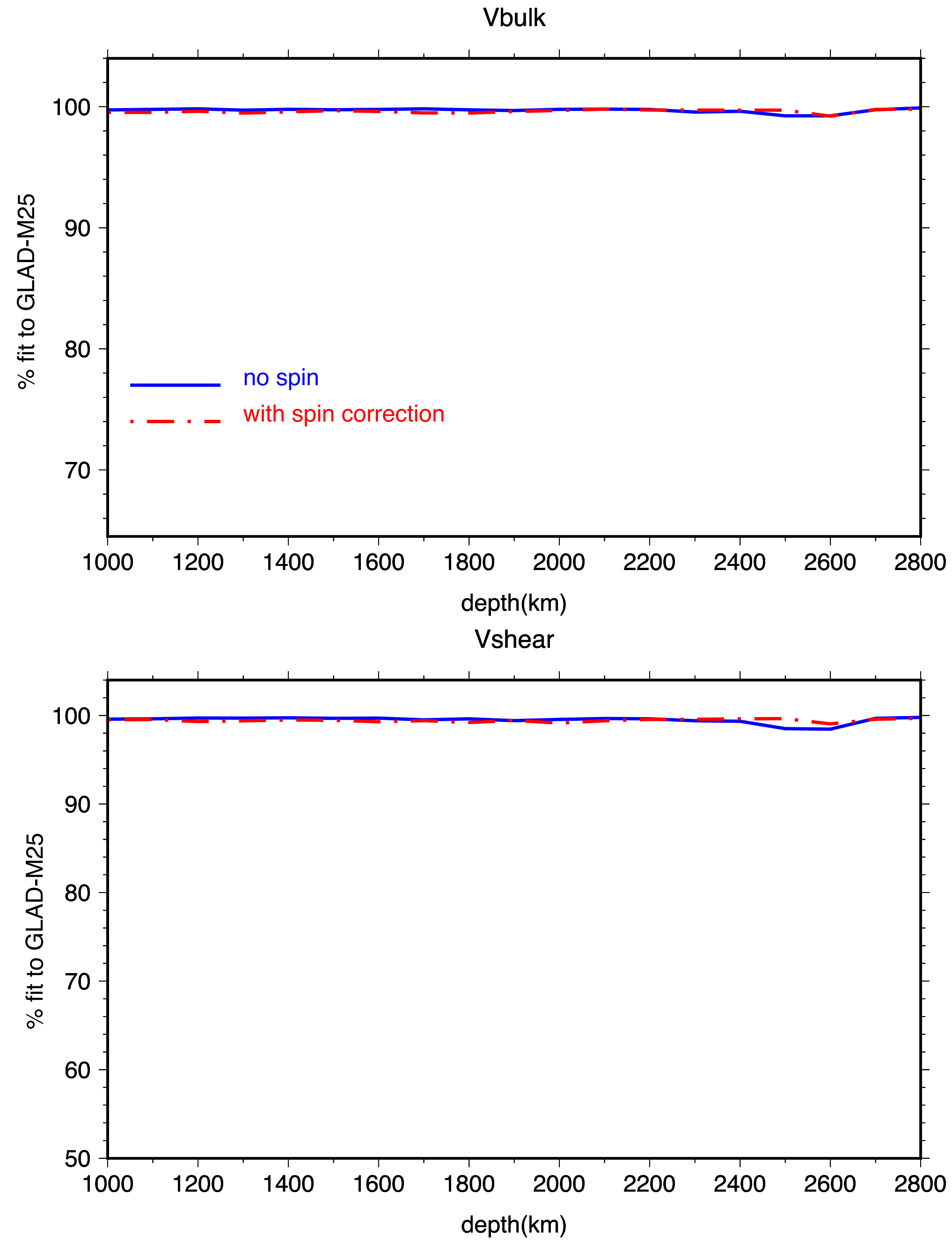

Fig. S7.

Comparison of fits to GLAD-M25 for Prior 2, with (red dashed line) and without (blue line) a spin transition. For clarity, results are shown for just one spin model: non-ideal solid solution with magnetic entropy. This spin model provides the most plausible temperature and composition gradients (see Figures S8-S12).

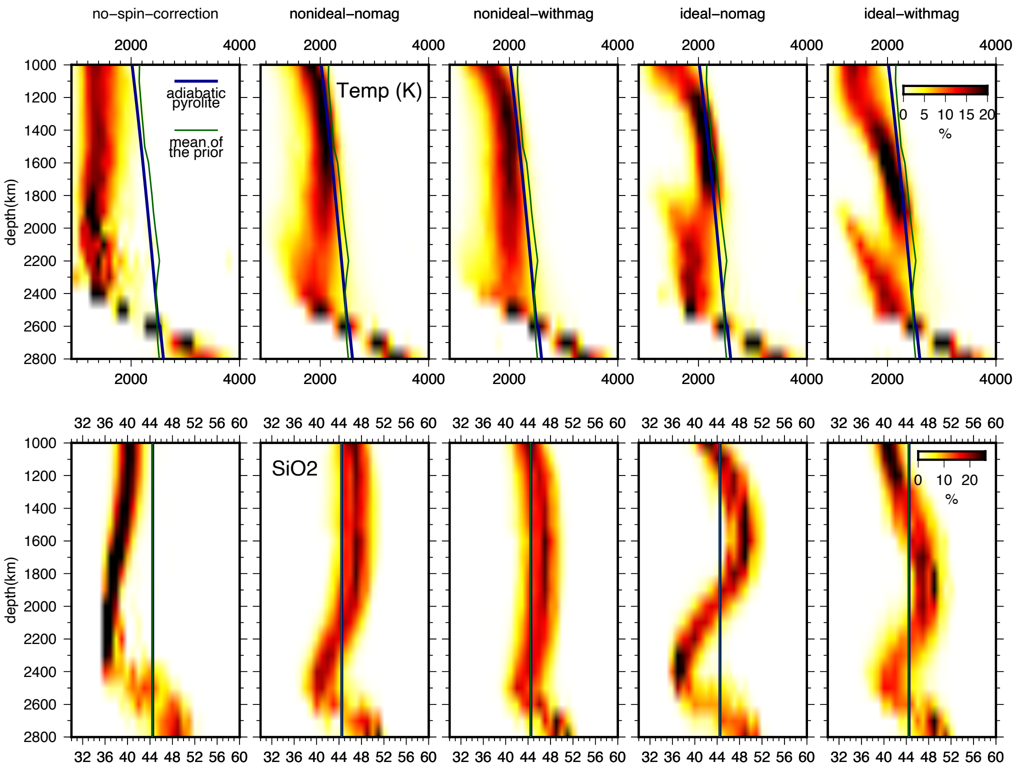

Fig. S8.

Density plots showing distributions of temperature (top row) and wt% SiO_2 (bottom row) as a function of depth, for models with variable, but restricted, chemical composition (Prior 3, Fig. S1). Adiabatic pyrolite with potential temperature 1573 K (blue line) and mean of the prior (green line) are shown for comparison. On the left, models without a correction to the wavespeeds for spin transition. These models are both cold and very Si-poor in the mid-mantle. Other four columns show the results for 4 different spin corrections. Models which include non-ideal solid solution give more reasonable temperature and compositional gradients, in particular the model with both non-ideal solid solution and a correction for magnetic entropy.

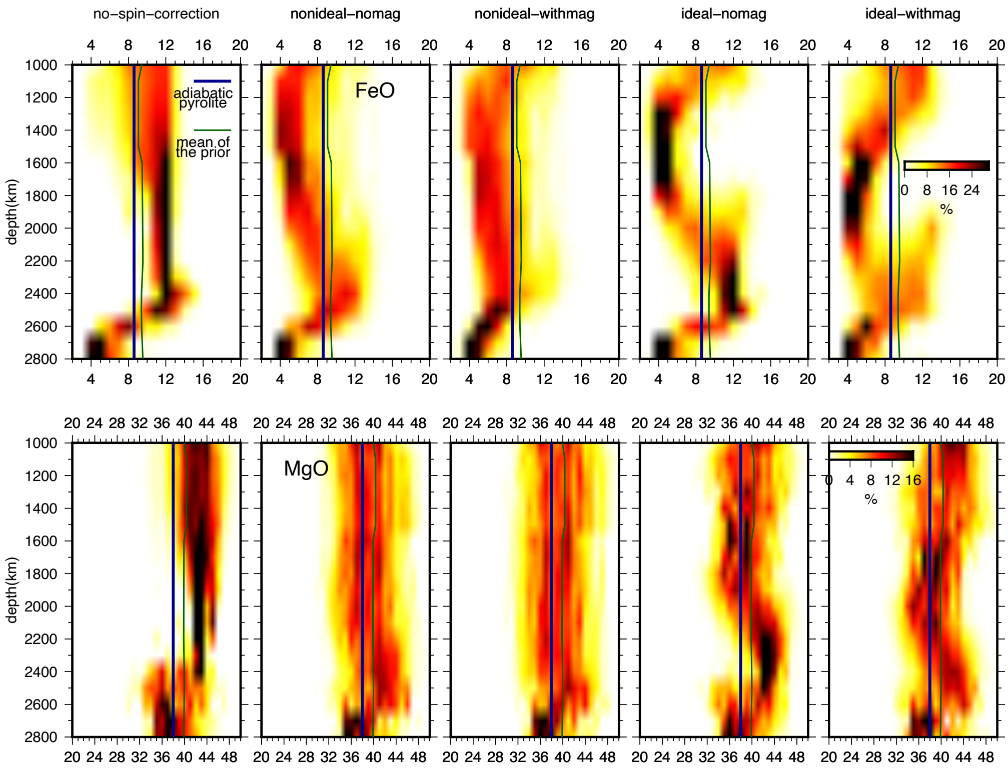

Fig. S9.

Density plots showing distributions of wt% FeO (top row) and wt% MgO (bottom row) as a function of depth, for models with variable, but restricted, chemical composition (Prior 3, Fig. S1). Pyrolite (blue line) and mean of the prior (green line) are shown for comparison. On the left, models without a correction to the wavespeeds for spin transition. The other four columns show the results for 4 different spin corrections. Models which include non-ideal solid solution give more reasonable compositional gradients, in particular the model with both non-ideal solid solution and a correction for magnetic entropy. All models show a decrease in iron towards the CMB which is likely driven by the need to increase the bulk wave speed (see main text for discussion).

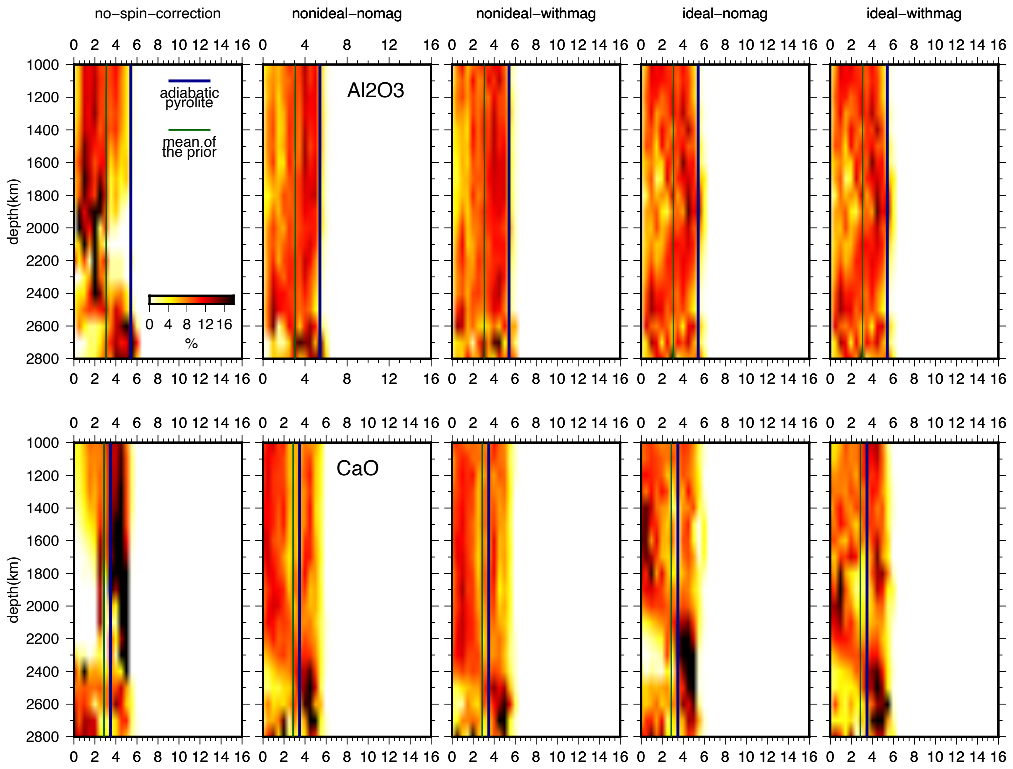

Fig. S10.

Density plots showing distributions of wt% Al_2O_3 (top row) and wt% CaO (bottom row) as a function of depth, for models with variable, but restricted, chemical composition (Prior 3, Fig. S1). Pyrolite (blue line) and mean of the prior (green line) are shown for comparison. On the left, models without a correction to the wavespeeds for spin transition. Other four columns show the results for 4 different spin corrections. With non-ideal solid solution the distributions are mostly broad and centred around the mean of the prior.

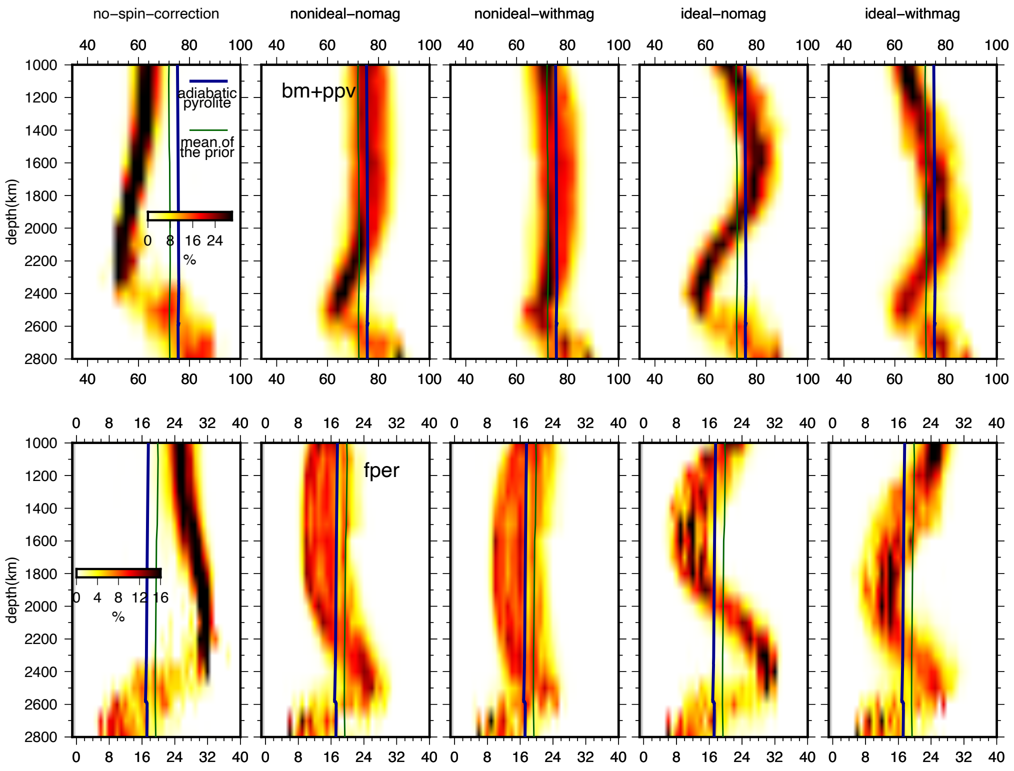

Fig. S11.

Density plots showing frequency distributions of minerology as a function of depth, for models with variable, but restricted, chemical composition (Prior 3, Fig. S1). On the top row, (Mg,Fe)SiO_3 (bridgmanite plus post-perovskite), and on the bottom row (Mg,FeO) ferropericlase. Pyrolite (blue line) and mean of the prior (green line) are shown for comparison. On the left, models without a correction to the wavespeeds for spin transition. The other four columns show the results for 4 different spin corrections. Models which include non-ideal solid solution give more reasonable vertical gradients, in particular the model with both non-ideal solid solution and a correction for magnetic entropy. Enrichment in silica towards the CMB is manifested in the mineralogy as an enrichment in (bridgmanite+post-perovskite) and depletion in ferropericlase. Without a correction for spin transition, the bridgmanite content is very low throughout the lower mantle.

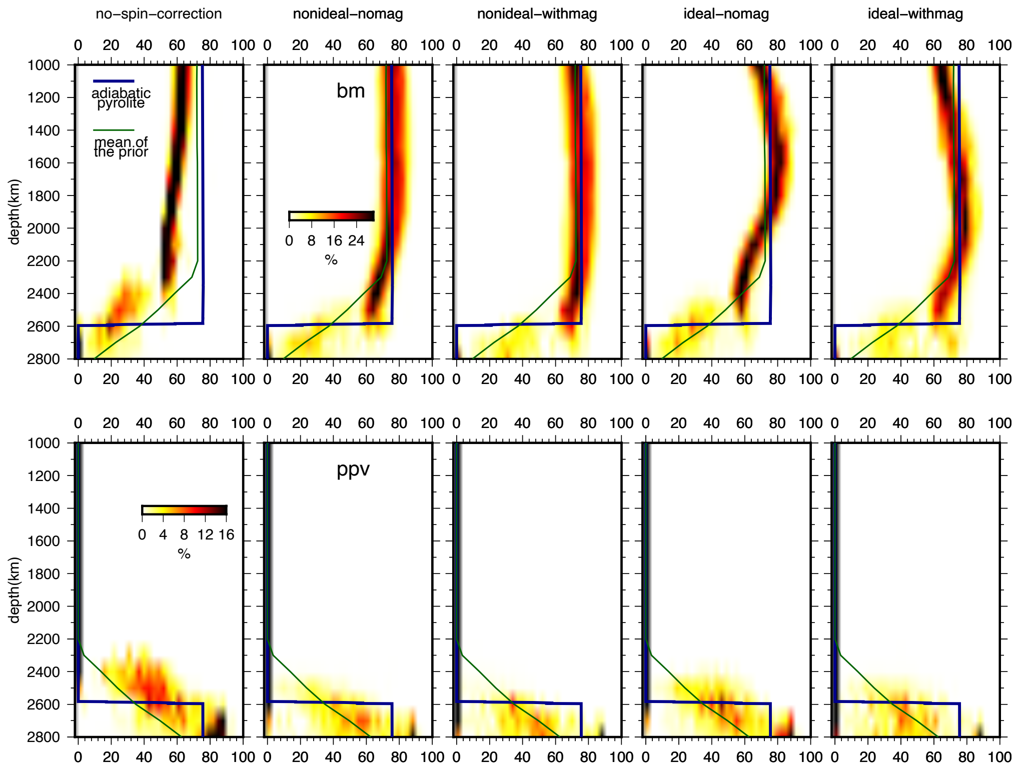

Fig. S12.

Density plots showing frequency distributions of minerology as a function of depth, for models with variable, but restricted, chemical composition (Prior 3, Fig. S1). On the top row, $(Mg,Fe)SiO_3$ bridgmanite (bm), and on the bottom row$(Mg,Fe)SiO_3$ post-perovskite (ppv). Pyrolite (blue line) and mean of the prior (green line) are shown for comparison. On the left, models without a correction to the wavespeeds for spin transition. The other four columns show the results for 4 different spin corrections. Without a spin transition, the bridgmanite content is very low throughout the mantle. With a spin transition, the average bridgmanite content is close to pyrolite. Models which include non-ideal solid solution give more reasonable vertical gradients, in particular the model with both non-ideal solid solution and a correction for magnetic entropy.

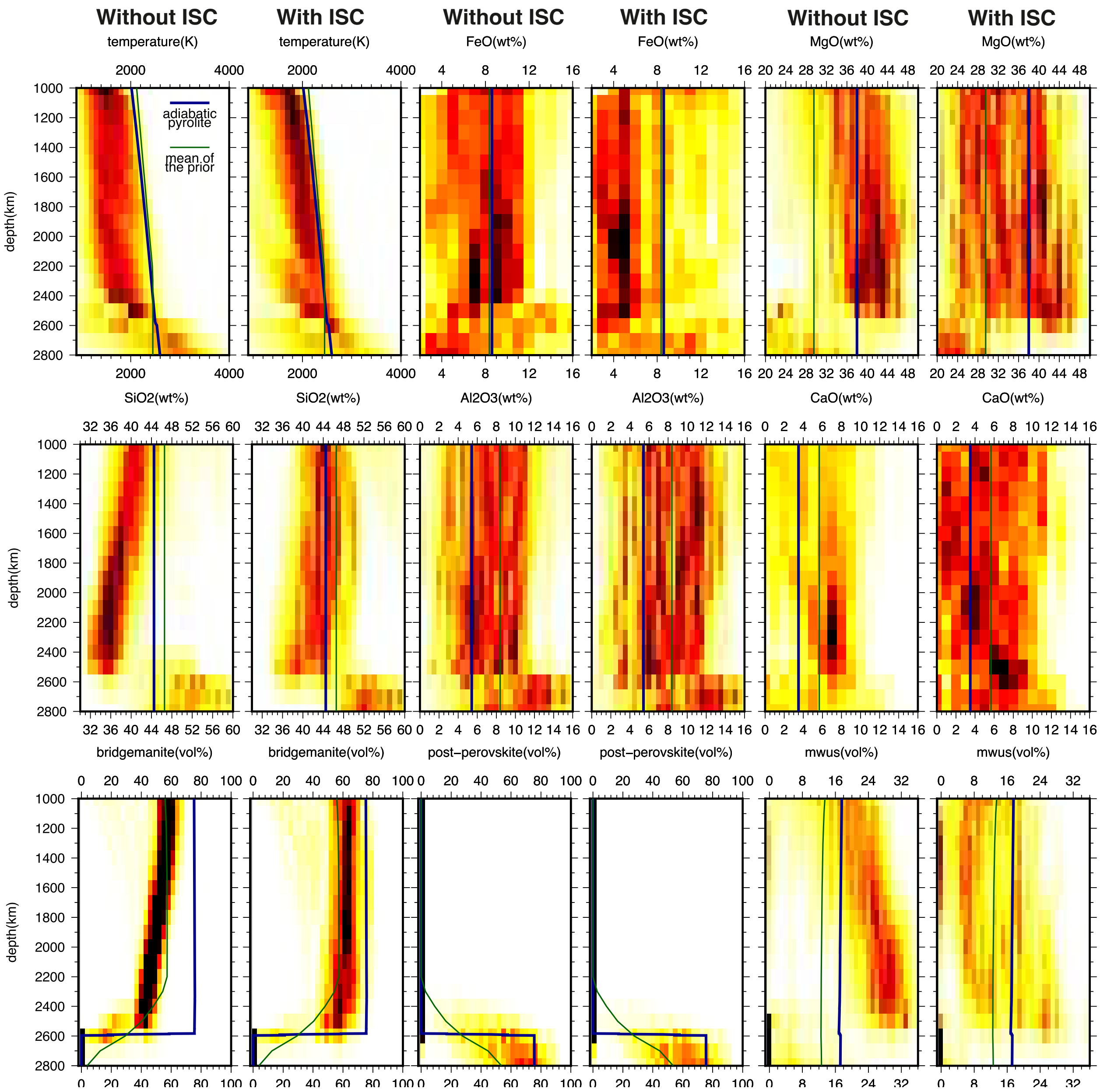

Fig.S13.

Density plots showing frequency distributions of temperature, bulk composition and mineralogy (Mg,Fe minerals) for the best-fitting set of models drawn from Prior 2 (Fig S1), as a function of depth. In each pair of plots, the left column is without a correction for spin transition in ferropericlase and the right is with a correction for spin transition. Due to the broad nature of the prior compared to Prior 3 (Figs S8-S12), the posterior distributions are correspondingly broader than those of Prior 3. Models from Prior 3 have bridgmanite contents closer to pyrolitic than Prior 2. The spin correction shown here is with non-ideal solid solution and a correction for magnetic entropy.

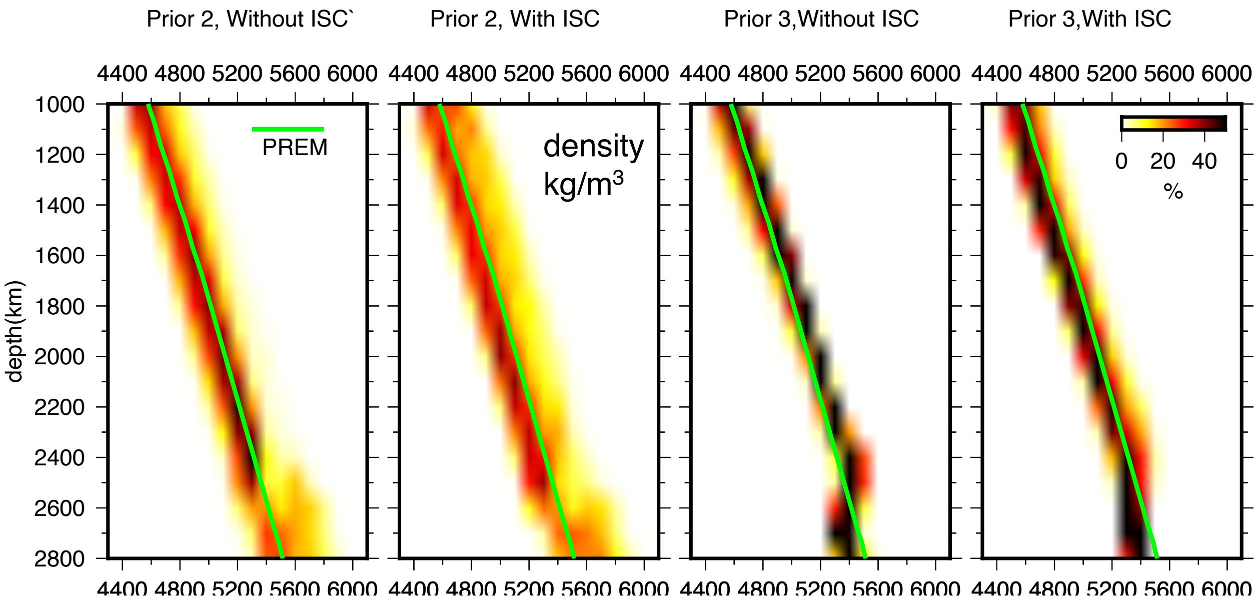

Fig.S14.

Frequency distributions of density (horizontal axis, kg/m^3) as a function of depth, with and without a spin correction. PREM is shown for reference with a green line. The density distributions correspond to the subset of thermochemical models selected from the stated Prior, by fitting bulk and shear wavespeeds to GLAD-M25. In D”, models drawn from Prior 2 (left two plots) follow the trend in PREM more closely than models drawn from Prior 3 (right 2 plots).

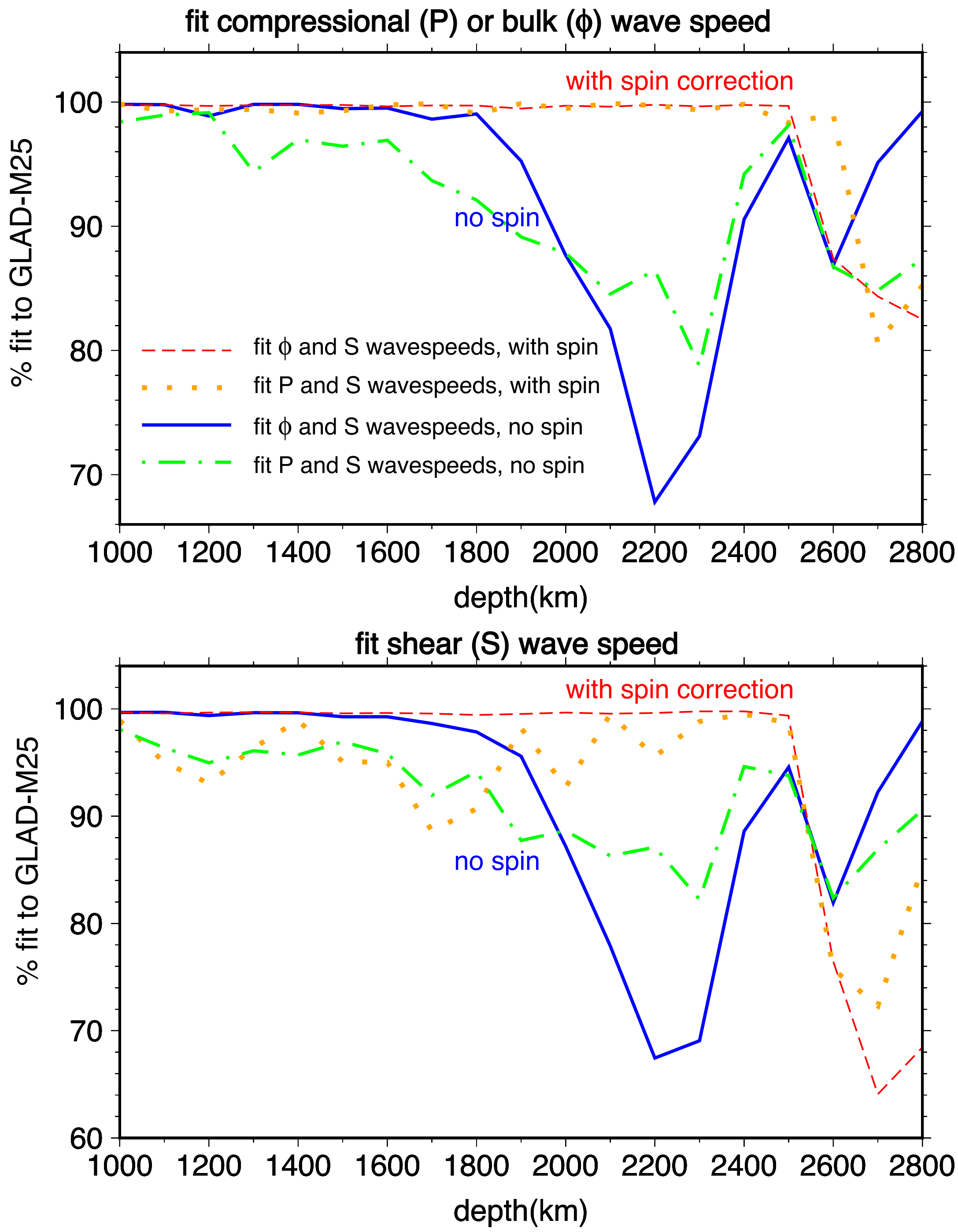

Fig. S15.

Comparison of the results when we fit bulk(ϕ) and shear(S) wave-speeds simultaneously (red dashed and blue solid lines) versus fitting compressional (P) and shear wave-speeds simultaneously (orange dotted and green dot-dashed lines). Top panel shows the fit to either V_P or V_ϕ as specified in the legend. Bottom panel shows the fit to V_S, having simultaneously fitted either V_P or V_ϕ as specified in the legend. The misfit of NOT including a spin transition in the mid-mantle becomes stronger when we consider bulk wave speed rather than compressional wavespeed (compare not only the blue with the green line, but also the difference between the blue and the red lines, versus the difference between the orange and the green lines).

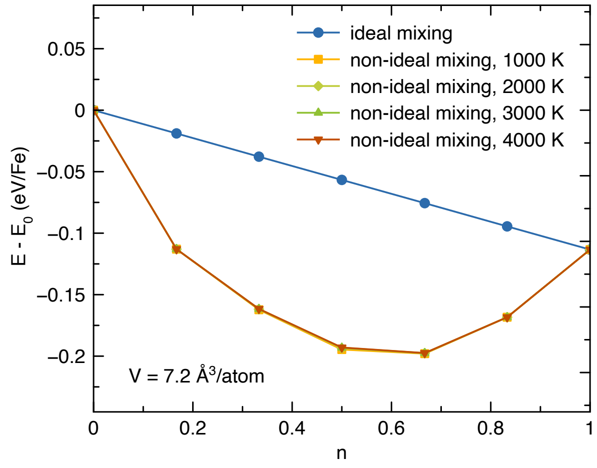

Fig. S16.

Static energy $E^{st}(V, T, n)$ per iron vs. *n* at constant *V* for x_{Fe}=0.1875.

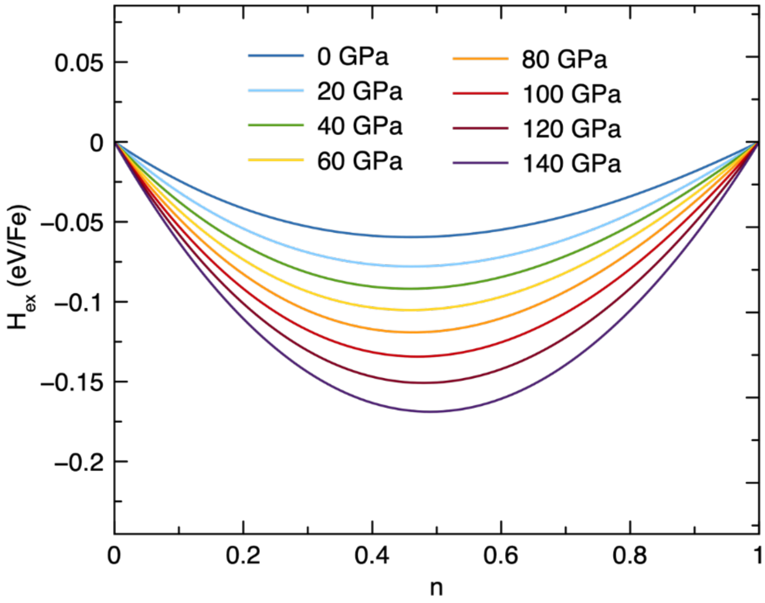

Fig. S17.

$H_{ex}(P, n)$ fit to a 3rd order polynomial in *n* as indicated in Eq. (7).

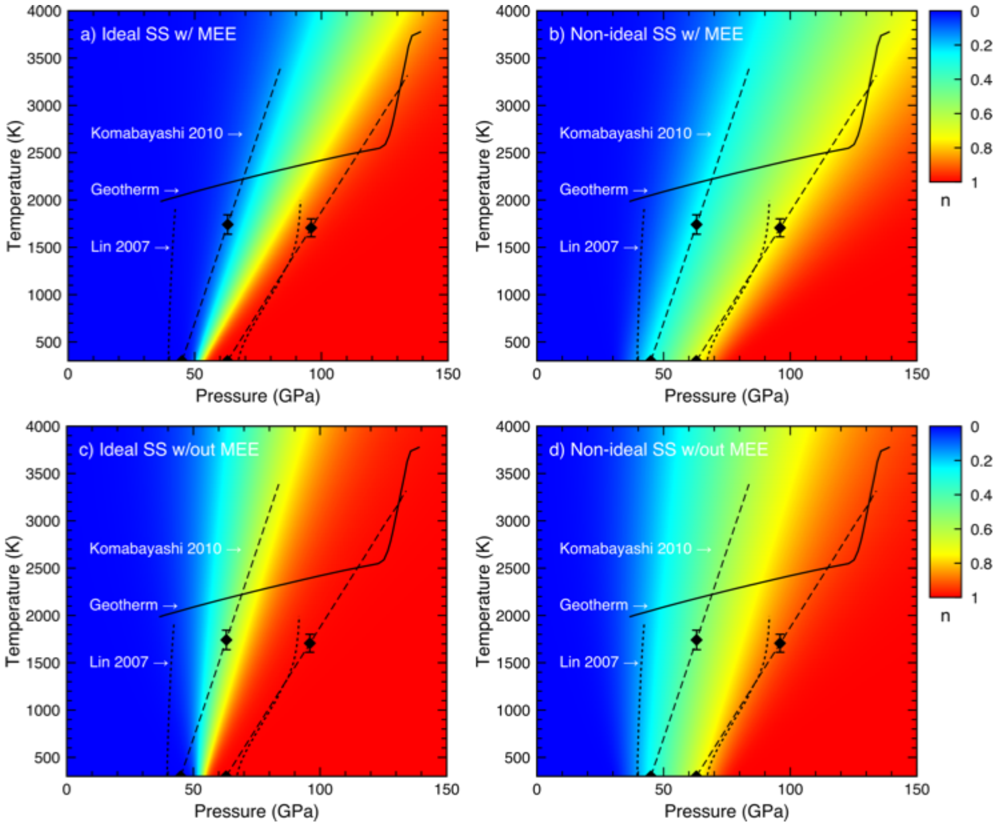

Fig. S18.

$n(P, T)$ for x = 0.1875 for four different thermodynamic models of the ISC in *fp*. (a) Ideal mixing between HS and LS states with magnetic entropy effects (MEE); (b) non-ideal mixing with MEE; (c) ideal mixing without MEE; (d) non-ideal mixing without MEE.

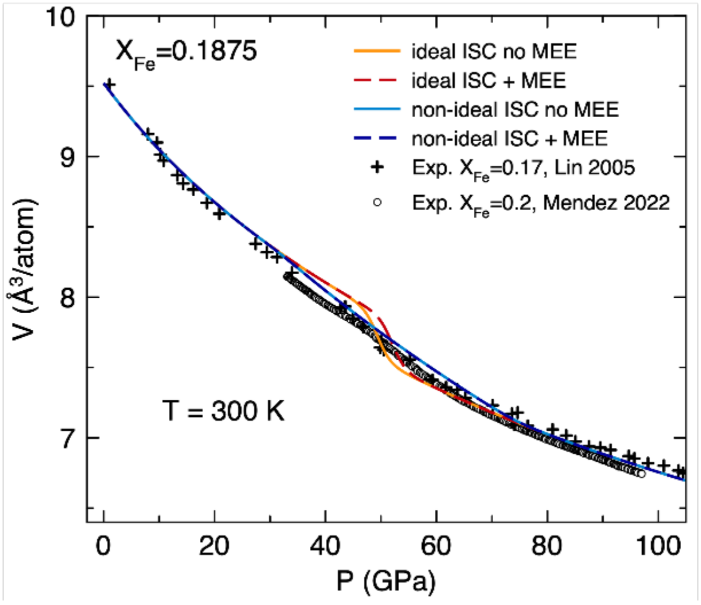

Fig. S19

300 K compression curves of the four thermodynamic models of *fp* with x = 0.1875 shown in Fig. S18.

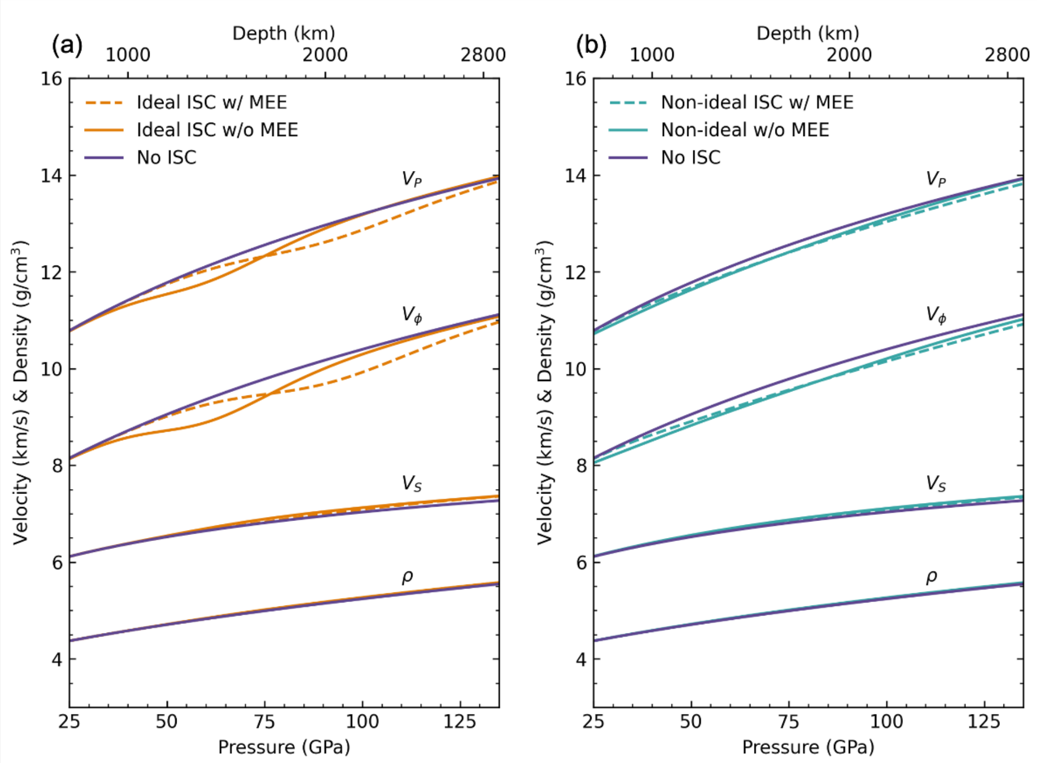

Fig. S20 – Comparison of velocities and densities of an aggregate with 63 vol% of $(Mg_{0.92}Fe_{0.08})SiO_3$ plus 37 vol% of $(Mg_{0.85}Fe_{0.15})O$ for different ISC models along the Brown and Shankland geotherm. (a) ideal HS/LS ferropericlase ISC, with and without MEE, and no ISC. (b) same but for a non-ideal SS model for the ISC.

		MgO		SiO2		FeO		Al2O3		CaO		Na2O	
		min	max	min	max	min	max	min	max	min	max	min	max
PRIOR 1: PYROLITE	uniform ranges	37.0	39.0	44.7	46.0	7.6	10.0	3.5	4.5	3.0	3.6	0.3	0.6
	after normalisation	36.7	39.4	44.0	46.8	7.6	10.0	3.5	4.6	3.0	3.7	0.3	0.6
PRIOR 2: VARIABLE	uniform ranges	5.0	75.0	50.0	65.0	3.0	18.0	0.0	18.0	0.0	14.0	0.0	4.0
COMPOSITION (BROAD)	after normalisation	4.8	53.9	30.0	78.0	1.9	20.8	0.0	20.8	0.0	15.9	0.0	5.4
PRIOR 3: VARIABLE	uniform ranges	33.0	52.0	42.0	51.0	4.0	15.0	0.0	6.0	0.0	6.0	0.0	0.5
COMPOSITION (RESTRICTED)	after normalisation	30.5	51.5	36.3	54.3	3.6	15.8	0.0	6.3	0.0	6.4	0.0	0.6

Table S1.

Compositional ranges of the three prior distributions plotted in Fig. S1. Oxides are given in wt %. We first draw random values for each oxide from uniform distributions specified by the limits given for “uniform ranges”. We then normalize these values so that the sum over the six oxides is 100 %. This has the effect of extending the ranges and making the distributions non-uniform.

depth (km)	PRIOR 1	PRIOR 2	PRIOR 3
1000	330000	672150	641272
1100	329999	754800	750000
1200	330000	754800	750000
1300	329999	754800	750000
1400	330000	754800	750000
1500	330000	754800	750000
1600	330000	665636	750000
1700	330000	663694	750000
1800	329999	660133	750000
1900	329999	636289	750000
2000	329999	628995	750000
2100	330000	624425	750000
2200	329999	617936	750000
2300	329999	637946	750000
2400	329999	653312	750000
2500	329999	650940	750000
2600	329999	640419	750000
2700	330000	622759	750000
2800	329999	601486	750000

Table S2.

Number of thermochemical models used to fit seismic data (GLAD-M25) after removing those which are thermodynamically unstable. Prior 1 (pyrolite) initially has 330,000 models per 100 km and Priors 2 and 3 initially have 750,000 models.

seismic period	1	s
frequency dependence (α)	0.274	
activation energy	286	kJ/mol
activation volume	1.2×10^{-6}	m^3/mol
Qref	312	
Tcore-mantle-boundary	3500	kJ/mol

Table S3.

Parameters used in correcting shear wave-speeds for anelasticity, based on Deschamps et al. (2019) [2]. Seismic period is the period at which GLAD-M25 is calculated.

Low spin fraction n	Space group of inequivalent configurations	Multiplicity
$\frac{1}{6}$	#123 P4/mmm	1
$\frac{1}{3}$	#123 P4/mmm	6
	#131 P4_2/mmc	6
	#139 I4/mmm	3
$\frac{1}{2}$	#123 P4/mmm	6
	#221 Pm-3m	2
	#47 Pmmm	12
$\frac{2}{3}$	#123 P4/mmm	6
	#131 P4_2/mmc	6
	#139 I4/mmm	3
$\frac{5}{6}$	#123 P4/mmm	1

Table S4. List of inequivalent HS/LS configurations for different values of n and x_{Fe}=0.1875.

References

1 Eggins, S. M., Rudnick, R. L. & McDonough, W. F. The composition of peridotites and their minerals: a laser-ablation ICP–MS study. *Earth and Planetary Science Letters* **154**, 53-71 (1998). https://doi.org:https://doi.org/10.1016/S0012-821X(97)00195-7

2 Deschamps, F., Konishi, K., Fuji, N. & Cobden, L. Radial thermo-chemical structure beneath Western and Northern Pacific from seismic waveform inversion. *Earth and Planetary Science Letters* **520**, 153-163 (2019). https://doi.org:https://doi.org/10.1016/j.epsl.2019.05.040
